# Diverticulite de Meckel d´origine ascaridienne chez l´enfant: à propos d´un cas

**DOI:** 10.11604/pamj.2021.39.92.29237

**Published:** 2021-06-01

**Authors:** Papa Alassane Mbaye, Mbaye Fall, Mohamed Salsabil Sabounji, Ndeye Aby Ndoye, Florent Tshibwid A Zeng, Ibrahima Bocar Wellé, Ndeye Fatou Seck, Gabriel Ngom

**Affiliations:** 1 Service de Chirurgie Pédiatrique, Hôpital d’Enfants Alber Royer, Université Cheikh Anta Diop, Dakar, Sénégal,; 2 Service de Chirurgie Pédiatrique, Hôpital Aristide Le Dantec, Université Cheikh Anta Diop, Dakar, Sénégal

**Keywords:** Diverticulite de Meckel, diverticule de Meckel, ascaris, ascaridiose, à propos d’un cas, Meckel´s diverticulitis, Meckel´s diverticulum, ascaris, ascaridiosis, case report

## Abstract

Nous rapportons l´observation d´un enfant âgé de 4 ans de sexe masculin, qui a été reçu pour un bourgeon ombilical congénital accompagné de saignements récents. L´examen physique retrouvait un bourgeon ombilical de couleur rosée, taché de sang sans fistule objectivée, d´environ 1,5 cm de diamètre. Une échographie abdominale a été demandée faisant évoquer un sinus de l´ouraque. L´exploration chirurgicale a mis en évidence un bourgeon ombilical communiquant en intra-abdominal avec un diverticule de Meckel à 90 cm de l´angle iléo-caecal hyperhémié, inflammatoire à l´intérieur duquel siégeait beaucoup d´ascaris. L´examen anatomo-pathologique de la pièce opératoire était en faveur d´une diverticulite. Ainsi, le diagnostic d´une diverticulite de Meckel d´origine ascaridienne a été retenu. Une résection-anastomose avec exérèse du bourgeon fut réalisée. Les suites opératoires étaient simples et après un recul de 6 mois.

## Abstract (en)

We here report the case of a 4-year-old male child presenting with congenital umbilical bud that, recently, had begun to bleed. Physical examination showed pinkish umbilical bud with blood stains but without any fistula, measuring about 1,5 cm in diameter. Abdominal ultrasound was performed, which suggested urachal sinus. Surgery revealed umbilical bud communicating, in the abdominal portion, with hyperemic, inflammatory Meckel´s diverticulum placed 90 cm away from the ileocaecal angle in which many ascaris were detected. Anatomo-pathological examination of the surgical specimen showed diverticulitis. Thus, the diagnosis of Meckel´s diverticulitis by Ascaris was retained. Resection and anastomosis with bud excision were performed. The postoperative course was simple even after a 6-month follow-up period.

## Introduction

Le diverticule de Meckel (DM) est le vestige d´une fermeture incomplète de la partie proximale du canal omphalo-mésentérique également appelé canal vitellin. Ce canal relie la vésicule vitelline à l´intestin primitif et se ferme généralement entre les sixièmes et neuvièmes semaines du développement embryonnaire [1]. Elle représente chez 2 à 3% de la population [1]. Dans la majorité des cas, le diverticule de Meckel reste latent et asymptomatique. Cependant, il peut se compliquer sous la forme d´une hémorragie digestive, d´une occlusion ou d´une diverticulite. Le risque de survenue de complication au cours de la vie est évalué à 2% [2]. Ces complications peuvent survenir tout au long de l´existence, mais particulièrement fréquentes chez l'enfant. Nous rapportons ce cas particulier d´un enfant de 4 ans présentant une diverticulite de Meckel d´origine ascaridienne.

## Patient et observation

Il s´agit d´un enfant âgé de 4 ans, de sexe masculin, qui nous a été adressé pour la prise en charge d´un bourgeon ombilical évoluant depuis la naissance. Le malade était initialement asymptomatique. Il avait présenté un jour avant son admission des saignements en nappe au niveau du bourgeon. Nous n´avions pas noté d´antécédents pathologiques médico-chirurgicaux particuliers ni de notion de consanguinité.

**Figure 1:**
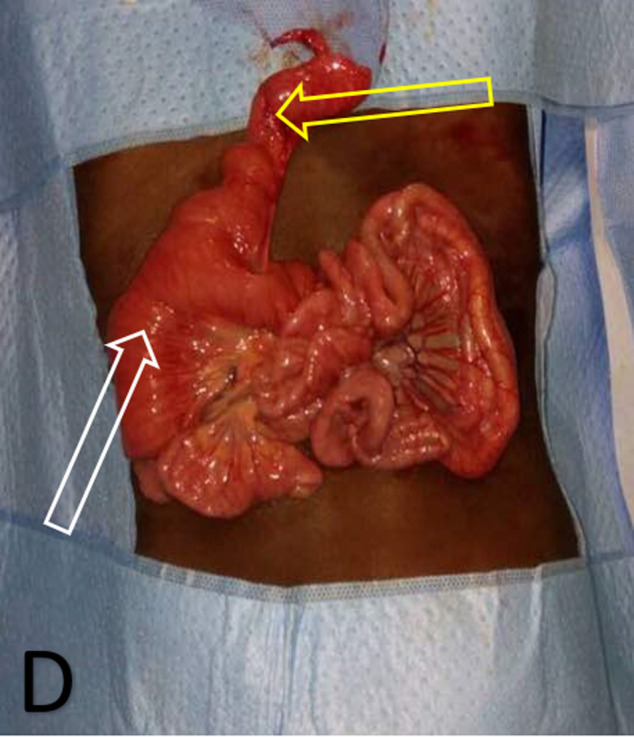
image peropératoire montrant le diverticule de Meckel; DM hyperémié, inflammatoire et augmenté de volume (flèche jaune); augmentation du volume de l´iléon distal au diverticule contenant des ascaris adultes (flèche blanche)

A son admission, l´enfant présentait un bon état général, des muqueuses conjonctivales de coloration normale, il était apyrétique avec un bon état de nutrition et d´hydratation. L´examen physique retrouvait un bourgeon ombilical de couleur rosé, taché de sang, sans fistule objectivée, d´environ 1,5 cm de diamètre. L´abdomen était souple sans masse palpable. La miction était normale. Le reste de l´examen était sans particularité. Devant ce tableau, une échographie abdominale fut réalisée montrant ainsi une formation ombilicale ovalaire sous-cutanée richement vascularisé sans rapport avec la cavité péritonéale faisant évoquer le diagnostic d´un sinus de l´ouraque. Une laparotomie exploratrice a été indiquée. Elle fut réalisée par voie d´abord sus-ombilicale. L´exploration chirurgicale a mis en évidence un bourgeon ombilical communiquant en intra-abdominal à un DM situé à 90 cm de l´angle iléo-caecal hyperhémié et inflammatoire (Figure 1).

**Figure 2:**
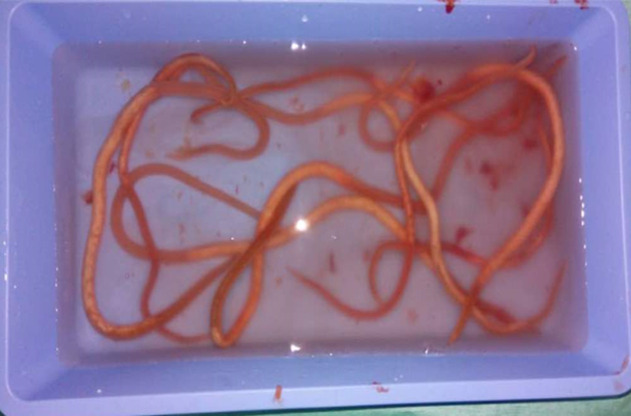
quelques ascaris extraits du DM et de l´iléon adjacent

Par ailleurs, nous avons noté la présence de vers d´ascaris adultes au nombre de 13 dans la lumière diverticulaire et des anses adjacentes (Figure 2). Une résection de 5 cm de part et d´autre du diverticule avec exérèse du bourgeon ombilical fut réalisée suivie d´une extraction des vers d´ascaris adultes et enfin une anastomose iléo-iléale termino-terminale. La pièce opératoire a été envoyée pour un examen anatomo-pathologique, lequel est revenu en faveur d´une diverticulite et n´a pas retrouvé d´hétérotopie muqueuse. Ainsi, nous avons retenu le diagnostic d´une diverticulite de Meckel d´origine ascaridienne. Le patient a bénéficié d´un traitement médical antiparasitaire en postopératoire immédiat. Les suites opératoires étaient simples. La sortie de l´hôpital était autorisée à J5 postopératoire. L´évolution était sans particularités après un suivi de 6 mois.

**Avis du patient:** les parents du patient ont déclaré être satisfaits du traitement entrepris, lequel a permis de significativement soulager leur enfant.

**Consentement éclairé:** les auteurs déclarent qu´un consentement écrit, pour publication, a été obtenu du père du patient.

## Discussion

Le DM est un reliquat embryonnaire que l´on retrouve sur le bord anti-mésentérique de l´iléon, situé habituellement à moins de 100 cm de la valvule iléo-caecale de Bauhin [1]. De plus, Il s´agit d´un vrai diverticule étant donné qu´il présente toutes les couches de la paroi intestinale [2]. Les complications du DM sont difficiles à diagnostiquer cliniquement et radiologiquement étant donné la faible spécificité des symptômes, des signes cliniques et des caractéristiques à l´imagerie. La fréquence du DM est difficile à apprécier et varie entre 0,3 à 4% selon les auteurs [3, 4].

Selon Kimberly [3], le diverticule de Meckel s´associe fréquemment avec certaines malformations. Chez notre patient aucune malformation n´a été notée. A ce jour aucun antécédent ne peut être retenu formellement dans cette pathologie. Toutefois, il n'est pas exceptionnel de retrouver un DM chez plusieurs membres d'une même famille et l'existence d'une notion de consanguinité suggèrent une certaine prédisposition familiale [5]. Dans notre cas aucun antécédent n´a été retrouvé. Sa prédominance est masculine, avec une nette prédominance masculine dans le groupe de DM compliqué [6-8].

Le DM est le plus souvent latent, mais peut être la cause de complications diverses qui constituent autant de circonstances de diagnostic [4]. La plupart du temps, il n´entraine aucun trouble, son existence n´étant révélée que fortuitement à l'occasion notamment d'une exploration systématique de l'intestin grêle mais il peut évoluer vers des complications dont la difficulté diagnostique est bien classique [2]. Les examens d´imagerie, notamment en l´absence de complication, sont peu contributifs. Le DM peut se compliquer sous la forme d´une hémorragie digestive, d´une occlusion ou d´une diverticulite (particulièrement fréquentes chez l'enfant) [9, 10]. La diverticulite reste une complication peu fréquente, cela s´explique par la rareté des formations lymphoïdes au niveau du DM et par sa communication large avec l'intestin [1].

Elle peut se voir à tout âge, elle touche principalement le garçon (sex-ratio: 12/1). C'est une complication qui reste rare chez l´enfant, 10% pour Saint-Vil *et al*. [6] et 11% pour Pellerin *et al*. [5]. Cependant, une infestation massive en Ascaris peut aboutir à une diverticulite de Meckel secondaire à l´incarcération des vers [9]. C´est le cas pour notre patient avec comme étiologie de cette diverticulite une ascaridiose intestinale. Cliniquement, elle peut mimer le tableau d'une appendicite [8, 10]. La particularité de notre cas réside dans le mode de révélation atypique par un bourgeon ombilical avec un saignement faisant évoquer une pathologie de l´ouraque ou du canal omphalo-mésentérique.

Chez notre patient, la confirmation diagnostique n´a été faite qu´en postopératoire après examen anatomo-pathologique de la pièce opératoire. Le traitement d´un diverticule de Meckel symptomatique est toujours une résection chirurgicale [5]. En ce qui concerne le choix de l´abord chirurgical, le débat persiste. La laparoscopie semble être une bonne approche diagnostique dans les cas où la clinique et la radiologie n´ont pas permis d´établir le diagnostic. Elle est également une bonne approche thérapeutique [2, 3]. Ceci reste vrai pour la prise en charge des diverticules perforés [4]. Cependant, la laparoscopie ne peut être définie comme l´abord chirurgical de référence dans le traitement des DM symptomatiques étant donné le faible nombre d´études réalisées sur le sujet [2]. Il convient d´utiliser en priorité la voie d´abord la moins invasive dans la mesure du possible. Mais les deux approches, la laparotomie et la laparoscopie, ont leurs limites. Le choix reste dépendant des conditions du patient et de l´expérience du chirurgien [7].

En termes de technique chirurgicale, il n´existe pas de consensus universel. Certains auteurs préfèrent réaliser une résection intestinale du segment contenant le diverticule afin d´éviter de laisser du tissu ectopique au niveau de la base de celui-ci. D´autres préconisent plutôt une diverticulectomie car celle-ci comporte un taux de morbidité plus faible [7, 8]. Cependant, dans le cas où une masse palpable est identifiable à la base du diverticule de Meckel, il faut veiller à ce que les marges de résection soient saines et donc préférer une résection segmentaire grêle. Par contre, en l´absence de masse palpable à la base du diverticule, une simple diverticulectomie peut être suffisante [2]. Dans notre cas, nous avons réalisé une résection du diverticule avec anastomose iléo-iléale en un temps du fait de l´absence d´examen histologique extemporané.

La leçon à tirer de ce cas est la présentation clinique atypique des pathologies du diverticule de Meckel et dans notre cas, la diverticulite. En milieux à faible revenus, la cause parasitaire devrait être envisagée.

## Conclusion

Les complications issues de la présence d´un diverticule de Meckel ne doivent pas être omises dans le diagnostic différentiel des douleurs abdominales. La prise en charge d´un diverticule de Meckel compliqué est toujours chirurgicale. L´origine ascaridienne d´une diverticulite doit être suspectée surtout dans nos régions d´endémie parasitaire.
